# Supplementary material for: A set of multi-entry identification keys to African frugivorous flies (Diptera, Tephritidae)
Source: Zookeys. 2014 Jul 24;(428):97–108. doi: 10.3897/zookeys.428.7366 (PMC4143993; doi:10.3897/zookeys.428.7366)
Supplement: Supplementary material 6 — Key to Ceratitis [file zookeys-428-097-s006.zip › SF6_ZooKeys_key to Ceratitis/key/SF6_ZooKeys_key to Ceratitis/Media/Html/Ceratitis curvata .htm]

Ceratitis (Pterandrus) curvata (Munro)


***Ceratitis*** **(*Pterandrus*) *curvata*
(Munro)**

*Pterandrus curvatus* Munro, 1937: 6.

Body
length: 3.97 (3.75-4.20) mm; wing length: 4.36 (4.20-4.70) mm.

Male

Head: Antenna yellow to yellowish orange. First flagellomere
three times as long as pedicel. Arista with short to moderately long rays,
basoventral rays shorter and sparser than basodorsal rays. Frons convex (rarely
more flat and slightly projecting at frontofacial angle); yellow, darker spots
near frontofacial angle and sometimes in center; with short scattered setulae
distinctly darker than frons. Frontal setae well developed. Face yellowish
white. Genal seta pale or more reddish; genal setulae pale, poorly developed.
Occiput on dorsal part yellowish brown to brown colored except along dorsal
margin.

Thorax: Postpronotal
lobe yellow, with brown spot. Mesonotum: ground color brown, occasionally
black, microtrichose areas silvery with ashgray shine, spots brownish black or
black except poorly developed sutural yellow spots, prescutellar yellow
markings separate. Scapular setae dark. Scutellum yellowish white, basally with
two merged dark spots, apically with three merged black spots, weakly incised,
extending to basal spots or almost so. Anepisternum on ventral half brown;
setulae pale, except ventral to anepisternal seta dark.

Legs:
Yellow except where otherwise noted; setation typical for subgenus, mixed pale
and dark. Foreleg: coxa with tuft of silvery setulae; femur yellow except
dorsally on basal half slightly darker, anteroventrally with dense comb of
silvery setulae along entire length; posteriorly with poorly developed bush of
long palish setulae along entire length, posterodorsal setulae and row of
dorsal setulae on distal half longer, basally darker setulae; ventral setae
pale. Midleg: femur anteriorly with short but conspicuous dense black pilosity;
along dorsal distal half with row of longer black flattened setulae, gradually
shortening towards apical end, with silvery patch on distal 0.33 (largely
obscured by dark pilosity); ventrally with dispersed dark setulae. Hindleg:
femur at apical 0.33 with longer setulae dorsally and ventrally.

Wing:
bands brownish. Marginal band forming continuous band with anterior part
of discal band; cubital band free; medial band absent; crossvein R-M proximal
to middle of discal cell. Apex of vein R_1_ distal to level of
crossvein R-M. Crossvein DM-Cu oblique anterobasally.

Abdomen:
Tergite 1 yellowish orange with brown patches across posterior margin; tergites
2 and 4 anteriorly brown, with silvery transverse band along� posterior half;
tergite 3 completely dark brown; tergite 5 orange brown. Setation and banding
typical for subgenus. Male epandrium in lateral view distinctly broadened;
posterior lobe of lateral surstylus with base broadened, apical end sharply
pointed.

Female

As
male except for the following characters: First flagellomere 2-3 times as long
as second, more orange colored. Genal seta and setulae dark, latter well
developed. Postpronotal spot more distinct. Legs without feathering; yellow
except femora brownish; pilosity and ventral setae forefemur dark. Oviscape
shorter than preabdomen. Aculeus at least 10 times longer than wide; tip flat
in lateral view, pointed, and with lateral margin straight.

(Description
after De Meyer & Freidberg, 2006)
